# Hydrodynamic modelling of traffic-related microplastics discharged with stormwater into the Göta River in Sweden

**DOI:** 10.1007/s11356-020-08637-z

**Published:** 2020-04-18

**Authors:** Mia Bondelind, Ekaterina Sokolova, Ailinh Nguyen, Dick Karlsson, Anna Karlsson, Karin Björklund

**Affiliations:** 1 Department of Architecture and Civil Engineering, Chalmers University of Technology, Sven Hultins gata 6, SE-412 96 Gothenburg, Sweden; 2 Sustainable Waste and Water, City of Gothenburg, Box 123, SE-424 23 Angered, Sweden; 3 Tyréns AB, Lilla Badhusgatan 2, SE-411 21 Gothenburg, Sweden

**Keywords:** Fate in receiving waters, Microplastic particles, Road run-off, Settling, Three-dimensional hydrodynamic model, Traffic-related emissions

## Abstract

Microplastics (MP) are transported from land-based sources from rivers to marine waters. However, there is currently little knowledge about MP fate from land sources to marine waters. Traffic is estimated to be one of the largest sources of MP; hence, stormwater is expected to be an important transportation route of MP to marine waters. The aim of this study was to investigate the effect of the size and density of tyre wear particles in road run-off on their fate in the Göta River in Sweden using hydrodynamic modelling. The model of the stretch of Göta River, Sweden’s largest river, passing through Gothenburg (Sweden’s second largest city) and out to the sea, was set up using MIKE 3 FM software. Literature data were used to define the MP characteristics: concentrations in stormwater, prevalent particle sizes, density of MP commonly occurring in road run-off and settling velocities. Results show that higher concentrations of MP are found on the south side of the river, compared with the north side, due to higher annual average daily traffic loads along the south side of the river. The mixing processes in the river and the MP concentrations were generally influenced by the vertical water density gradient caused by saline water from the Kattegat strait. While most MP with higher density and larger size settle in the river, smaller MP with density close to 1.0 g/cm^3^ do not settle in the river and therefore reach the Kattegat strait and the marine environments. Further research is needed to describe the fate and transport of microplastics in the stormwater system, including treatment facilities, i.e. biofouling, aggregation, degradation and/or further fragmentation and settling.

**Electronic supplementary material:**

The online version of this article (10.1007/s11356-020-08637-z) contains supplementary material, which is available to authorized users.

## Introduction

Plastic pollution is present in most bodies of water on the planet; consequently, this has become one of our most serious global environmental problems (Shahul Hamid et al. 2018). Small plastic fragments are released into the environment from plastic manufacturing and other industrial applications, or when plastic objects are worn or degraded into smaller fragments once deposited in the environment. These small plastic fragments, called microplastics (MP), are usually defined as being smaller than 5 mm (Anderson et al. 2016). They are small enough to be ingested by marine biota and transferred through the food chain (Barboza and Gimenez 2015). As a result, MP have been detected in organisms at all levels of the marine food chain. Ingestion of MP may lead to adverse physical effects on biota, e.g. reduction in feeding or false satiation, and toxic effects from released hazardous substances added to the polymers (Wright et al. 2013; Baldwin et al. 2016). Due to their chemical structure, MP can absorb and concentrate hydrophobic persistent organic pollutants (POPs) present in water, e.g. polychlorinated biphenyls (PCBs) and polycyclic aromatic hydrocarbons (PAHs); also, metals and pathogens have been shown to sorb to MP (Baldwin et al. 2016; Hong et al. 2017; Anderson et al. 2016; Rocha-Santos and Duarte 2015). Microplastic exposure studies are limited, and there are large knowledge gaps in the research field, for example, the effects on aquatic ecosystems; however, new insights are continuously gained (SAPEA 2019).

It is estimated that 10% of the annual production of plastics (approximately 350 t in 2014 [Auta et al. 2017; Boucher and Friot 2017]) ends up in our oceans and that 70–80% of marine MP originate from land-based sources, transported mainly via rivers (Horton et al. 2017a, b). The global-scale models (Lebreton et al. 2017; Schmidt et al. 2017; van Wijnen et al. 2019) emphasise large spatial variations in MP inputs from rivers into oceans. Siegfried et al. (2017) estimated that the highest proportion—42%—of the total MP load into European rivers originates from road-related MP, transported with stormwater, whereas polymer-based textiles, transported with wastewater, account for 29% of the MP load. Sources of MP in road run-off include abrasion of tyres, which contain synthetic rubber (often styrene-butadiene rubbers), bitumen, road markings, vehicle body parts and littering (Kim et al. 2006; Magnusson et al. 2016; Bläsing and Amelung 2018). Tyre debris is identified as a major source of MP in marine environments: 28% of the global release of MP to oceans is assumed to be released from tyres (Boucher and Friot 2017). Tyre rubber contains several chemicals of environmental concern, such as PAH and Zn, and leachate from tyre particles has shown ecotoxicological effects on aquatic organisms (Wik and Dave 2009; Capolupo et al. 2020).

Microplastics transported with stormwater from road surfaces will either sink or accumulate in sediments or be transported through the stormwater system to receiving waters. Particle characteristics, such as shape, diameter and density, are determinant of MP fate in the stormwater system, including treatment facilities, and the particles’ transport into receiving waters (Avio et al. 2017). Turbulence in sewers and sediment traps (e.g. ponds and settling tanks) can cause resuspension of high-density MP and redistribution throughout the water column, leading to further transport of high-density MP into receiving waters (Anderson et al. 2016; Magnusson et al. 2016). Research shows that MP may adhere to mineral particles, which exhibit higher density than many polymers (Corcoran 2015). Also, particle density can change due to leaching of additives, and MP are subject to biofouling by bacteria, algae and other organisms, causing increased particle density (Anderson et al. 2016; Avio et al. 2017; Tsang et al. 2017). This creates an opportunity for lighter particles, which would not settle on their own but to settle through aggregation or biofouling. In addition, studies have shown that the development of a biofilm renders plastic particles less hydrophobic, which enhances their sinking (Lobelle and Cunliffe 2011).

Advances have been made in the area of transport modelling of particles in watersheds and water sources. Unice et al. (2019) modelled the spread of MP in the watershed using mass balance as a basis. To define the MP distribution in the water column, Vermeiren et al. (2016) designed conceptual models for MP transport in estuaries; it was suggested that physical and chemical processes affecting the MP fate and transport, e.g. the effect of wind or high UV exposure, should be investigated further. In an estuary model, which accounted for advective transport, aggregation, sedimentation-resuspension, degradation, presence of biofilm and sediment burial of MP, Besseling et al. (2017) demonstrated that particle size had a major effect on the fate and accumulation of MP. Although complex models which include many processes were derived, neither of these studies accounted for the effects of the time-dependent three-dimensional flow regime in the estuary.

Although traffic-related stormwater is expected to be a major transportation route of MP into receiving freshwater bodies and subsequently into marine waters (Siegfried et al. 2017), little is known about the fate and transport of MP in general, and of traffic-related MP in particular, in rivers; and this study aims to fill some of the knowledge gap. The aim of this study was to investigate the effect of the size and density of tyre wear particles in road run-off on their fate in the Göta River in Sweden using hydrodynamic modelling. To our knowledge, this is one of the first studies of the fate and transport of tyre wear particles, originating from stormwater discharges, in a receiving river using a time-dependent three-dimensional hydrodynamic model.

## Methods

### Study area

Only 8% of the stormwater volume in Sweden is currently treated, either in wastewater treatment plants (4%) or in local treatment facilities (4%) (Magnusson et al. 2016). This implies that the majority of road-related MP in Sweden is directly discharged into receiving waterways. In Sweden, the annual emissions of MP from rubber tyres are estimated to be approximately 13,000 t (Magnusson et al. 2016). The Göta River in south-west Sweden was chosen as a case study, as it is the largest river in Sweden, both in terms of catchment area (50,000 km^2^) and discharge (average 570 m^3^/s). Also, the Göta River passes through Gothenburg, the second largest city of Sweden (approximately 550,000 residents) and a transportation hub, with a major port (Scandinavia’s largest); approximately 120,000 persons commute into the city every day. Hence, the river is expected to be a major discharge point of MP into Swedish marine waters. The studied area in Gothenburg includes everything from residential streets with less than 1000 AADT to industrial areas with a higher abundance of heavy-load vehicles and arterial highways with up to 100,000 AADT. Included in the model are areas drained to separate stormwater sewers; hence, stormwater discharged into the river and its tributaries without prior treatment. Areas drained to a combined sewer system are not included in the model, as this stormwater is directed to the sewage treatment plant located at the mouth of the Göta River.

### Model of the Göta River

**Fig. 1:**
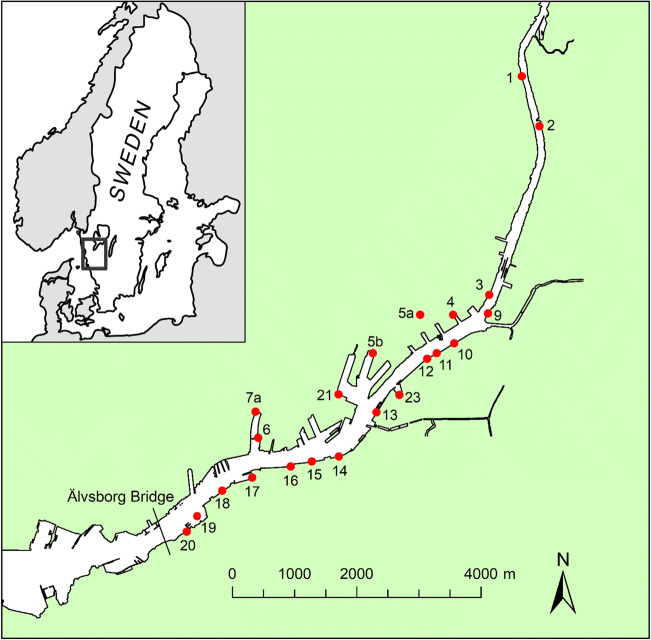
The modelling domain of the river with 22 stormwater discharge points (more information about the stormwater discharge points is provided in Table 2; for visualisation purposes, the letters “GÄ” in the point numbering were omitted in the figure). The Älvsborg Bridge is situated close to the mouth of the river where the river enters the Kattegat strait

In a previous study (Tyréns 2016), a hydrodynamic model was set up using MIKE 3 FM (MIKE Powered by DHI) software to describe the velocity field in the Göta River. The modelling domain included a 16-km stretch of the Göta River (Fig. 1), and the domain was approximated using a 3D flexible computational mesh: resolution 20–30 m in the horizontal and 1 m in the vertical direction. Input data to the hydrodynamic module included bathymetry (Fig. S1), water flows in the river (Fig. S2) and its tributaries (Fig. S3), water level in the Kattegat strait (Fig. S2), salinity and temperature at the model boundaries (Table S1) and wind conditions (Fig. S4). This hydrodynamic model of the Göta River was validated (Fig. S5) and then used to simulate the microbial water quality (Tyréns 2016) and the concentrations of benzo[a]pyrene and copper (Björklund et al. 2018) in the river. In this study, the model was further implemented to study the fate and transport of traffic-related MP released with stormwater into the river. The fate and transport of MP were simulated using the water quality module ECO Lab (MIKE Powered by DHI) coupled to the hydrodynamic model. In the ECO Lab module, it was assumed that MP are inert and their fate and transport in the river is governed solely by the advection-dispersion and settling processes.

The hydrodynamic model of the Göta River (Tyréns 2016) generally demonstrated higher water velocities in the surface layers in the middle of the river, while the velocity decreased towards the sides of the river. The residence time of the water was short in the middle section of the river but much longer in the harbours. The study showed that the average velocity for the period July–August was 0.25–0.45 m/s at the surface in the middle of the river. This means, for example, that the transport time between discharge point 23 (the Göta River Bridge) and the Älvsborg Bridge is about 3–5 h (Fig. 1). On the other hand, the residence time in the harbour Frihamnen (see discharge points 5b and 21 in Fig. 1) is about 1 d.

### Microplastics from traffic and roads

In this study, MP from traffic and roads were studied, as these are considered to be among the most important sources of MP in stormwater (Magnusson et al. 2016; Siegfried et al. 2017). An inventory of sources in the city of Gothenburg identified tyre rubber as the most important MP source (520 t per year), followed by artificial turf (35 t per year) and road markings (20 t per year) (Göteborgs Stad 2019). This is in agreement with a study by Kole et al. (2017) estimating the per capita wear and tear from tyres to range from 0.23 to 4.7 kg/year, with a global average of 0.81 kg/year. Similarly, a study of MP in road dust in Norway estimated the emissions of tyre wear to 7080–9600 t per year, whereas road wear particles (polymers added to bitumen and road marking paints) were estimated to be 120–210 t per year (Vogelsang et al. 2018). However, knowledge on the abundance of microplastics in stormwater is still limited, and very few monitoring studies exist to date.

Plastics with densities higher than water include the polymers polyvinylchloride (PVC) and polyethylene terephthalate (PET) and also tyre wear particles; common low-density polymers are, for example, polyethylene (PE) and polypropylene (PP) (Avio et al. 2017; Hanvey et al. 2017; Vogelsang et al. 2018). From the inventories of MP emissions to stormwater (Göteborgs Stad 2019; Vogelsang et al. 2018) and performed monitoring studies (Jannö 2016; Jönsson 2016; Trafikkontoret Göteborgs Stad 2018), it can be assumed that the majority of MP in road run-off are tyre wear particles. Tyre wear MP are generated during driving and appear mainly as cylindrical or spindle-shaped particles with rough surfaces. The particles typically contain a mix of tyre tread and road surface material, which explains the high density—usually 1.7 to 2.1 g/cm^3^—compared with the density of pure tread which is 1.15–1.18 g/cm^3^ (Vogelsang et al. 2018). Research reviewed by Vogelsang et al. (2018) suggests that tyre wear particles are in the micro-size scale, and the gross load is found in the size range of 50 to 100 μm. The typical size interval for these particles is 30–400 μm (Jannö 2016), 20–100 μm (Trafikkontoret Göteborgs Stad 2018) and 20–300 μm (Jönsson 2016) (Table 1). The maximum concentration of 1050 microplastic particles per litre (MP/L) stormwater measured by Jannö (2016) was measured in stormwater collected in the most highly trafficked area (approximately 100,000 vehicles/day) in the city of Gothenburg (Table 1). The magnitude of the concentrations was confirmed by a more recent study of MP in stormwater from another urban area in Gothenburg, where over 1500 MP/L was detected (Trafikkontoret Göteborgs Stad 2018). In stormwater ponds, even higher concentrations of particles (4267 MP/L) were measured (Jönsson 2016).

**Table 1:** Abundance and characteristics of microplastic particles detected in stormwater in Sweden

Reference	Stormwater source	Material	Investigated size fraction(s) (μm)	Number of particles (MP/L) per size fraction
Jannö (2016)	Road run-off, approx. 100,000 vehicles/day (Gothenburg, Sweden)	Synthetic fibres	30–400	291–500
		Black particles (tyre rubber and asphalt)	30–400	45–1050
Trafikkontoret Göteborgs Stad (2018)	Road run-off, approx. 20,000–50,000 vehicles/day (Gothenburg, Sweden)	Synthetic polymers	> 100	2–10
			20–100	50–160
		Road particles (tyre rubber and asphalt)	> 100	1–2
			20–100	1500–1600
Jönsson (2016)	Stormwater ponds (*n* = 3), run-off from mixed catchment areas (Sweden)	Microplastics	> 300	7.5∙10^−3^–19∙10^−3^
			20–300	5.4–10
		Black particles (tyre rubber, asphalt and/or combustion)	> 300	Not analysed (too few)
			20–300	220–4267

### Simulated scenarios

The hydrodynamic conditions in the river were simulated for two periods in 2015: 5–13 July (period 1) and 24 July–1 August (period 2). The total precipitation was 27 and 57 mm during periods 1 and 2, respectively. Precipitation occurred during one rain event for period 1 and during two rain events for period 2 (Fig. S6). As the hydrodynamic model of the Göta River is computationally expensive due its fine spatial resolution, these two periods were selected to represent the fate and transport of traffic-related MP released with stormwater into the river.

In our model, the discharges of stormwater containing traffic-related MP were included. The discharges of stormwater varied in time and corresponded to the volumes caused by the rain events at each discharge point. In total, 22 points representing discharges from separate stormwater sewers were located along the river (see Fig. 1 for locations and Figs. S7 and S8 for discharged volumes during the studied periods and the entire year 2015, respectively). The discharge point GÄ10 corresponds to the catchment area where the actual MP concentration was measured (Jannö 2016). Therefore, the concentration of 1050 MP/L measured by Jannö (2016) was assumed for discharge point GÄ10. For the other discharge points, the MP concentrations were calculated from the annual average daily traffic (AADT) in each corresponding catchment area (City of Göteborgs Stad 2019), in proportion to GÄ10 (Table 2).

**Table 2:** Characteristics of the catchment areas drained to the 22 stormwater discharge points (see Fig. 1) and assumed concentrations of microplastic particles (number of particles/L) in stormwater from each point

Discharge point^a^	Land use in catchment area	Traffic load compared with GÄ10	MP/L
GÄ1	Industrial/commercial, highway E6	0.80	840
GÄ2	Industrial/commercial, highway E45	0.03	33.3
GÄ3	Industrial/commercial, highway + tunnel	0.25	265
GÄ4	Industrial/commercial, highway	0.55	574
GÄ5a	Industrial/commercial, highway	0.08	80.2
GÄ5b	Industrial/commercial	0.03	35.9
GÄ6	Dense urban	0.02	23.9
GÄ7a	Dense urban	0.02	23.9
GÄ9	Industrial/commercial, highways E45/E6/E20	0.29	307
GÄ10	Industrial/commercial, highways E45/E6/E20	1	1050
GÄ11	Industrial/commercial, highway E45	0.93	982
GÄ12	Industrial/commercial, highway E45	0.93	982
GÄ13	Downtown area	0.04	39.3
GÄ14	Downtown area, highway E45	0.73	768
GÄ15	Downtown area, highway E45	0.81	853
GÄ16	Downtown area, highway E45	0.84	878
GÄ17	Downtown area, highway, industrial/commercial	0.78	821
GÄ18	Downtown area, highway, industrial/commercial	0.74	781
GÄ19	Dense urban, highway	0.76	794
GÄ20	Dense urban, highway	0.25	266
GÄ21	Dense urban	0.15	155
GÄ23	Downtown area	0.04	39.3

^a^The numbering of the points includes the letters “GÄ” for consistency with our earlier studies

To evaluate the distribution of MP in the Göta River and to clearly illustrate the effect of settling velocity, three scenarios for three different types of MP, in terms of particle size and density, were simulated. In Scenario 1, i.e. the non-settling scenario, the particles were assumed to be suspended in the water column; this would be the case for very small particles, e.g. very small tyre wear particles or plastic particles of low density, as many polymers have a density of < 1.0 g/cm^3^. In Scenario 2, the transport and settling of small and lightweight tyre wear particles, as reported in previous studies (Table 1), were simulated: Particle size 20 μm and density 1.7 g/cm^3^ were assumed. In Scenario 3, the transport and settling of average tyre wear particles were simulated: Particle size 75 μm and density 1.9 g/cm^3^ were assumed (average size and densities according to Vogelsang et al. 2018; Table 1). The settling velocities for particles assumed in Scenarios 2 and 3 were calculated using Stokes’ law (Eq. 1). In this calculation, the following assumptions were made: fresh water in the river and water temperature of 15 °C; the particles were assumed to be spherical although tyre wear particles appear to be cylindrical or spindle-shaped (Vogelsang et al. 2018).

1 $$ v=\frac{g\ {d}^2\ \left({\rho}_{\mathrm{p}}-{\rho}_{\mathrm{w}}\right)}{18\ \nu\ {\rho}_{\mathrm{w}}} $$

where *v* is the settling velocity (m/s), *g* is the acceleration of gravity (m/s^2^), *d* is the particle diameter (m), *ρ*_p_ is the density of particle (g/m^3^), *ρ*_w_ is the density of water (g/m^3^) and ν is the kinematic viscosity of medium (m^2^/s).

The settling velocities for Scenarios 2 and 3 were 1.3∙10^−4^ m/s and 2.4∙10^−3^ m/s, respectively.

In all scenarios, the effect of discharges from 22 stormwater points in the city was evaluated. All discharges were assumed to occur at the surface of the river. Due to their small size, the particles were assumed not to have any effect on the flow of the Göta River.

## Results

In this study, MP from traffic and roads were studied, as these are considered to be among the most important sources of MP in stormwater (Magnusson et al. 2016; Siegfried et al. 2017). An inventory of sources in the city of Gothenburg identified tyre rubber as the most important MP source (Göteborgs Stad 2019). The calculated amount of MP released with stormwater from the studied discharge points along the river during the year 2015 varied from 10^8^ to 10^12^ MP between the discharge points, and the highest amounts of MP were released from GÄ1, GÄ11 and GÄ14 (Fig. S8).

The distribution of MP in the Göta River during the rain events was simulated using the three-dimensional hydrodynamic model, and the effect of particle size and density, and hence settling velocity, on the MP distribution was evaluated. The three simulated scenarios differed in terms of the amount of MP that settled in the river compared with the amount of MP that reached the mouth of the river and consequently the sea. In Scenario 1, due to the assumption of no settling, all MP released with stormwater discharges passed through the river and reached the sea; in total, around 7.7∙10^10^ and 1.6∙10^11^ MP reached the sea during the simulated periods 1 and 2, respectively. In Scenario 2, 28% and 34% of all MP were discharged to the sea during periods 1 and 2, respectively, while the remaining portion of the particles settled in the river. In Scenario 3, almost all particles settled in the river and did not reach the sea; only 1.1∙10^6^ and 5.5∙10^6^ MP passed through the river cross-section at the Älvsborg Bridge during periods 1 and 2, respectively (see Fig. 1 for location).

**Fig. 2:**
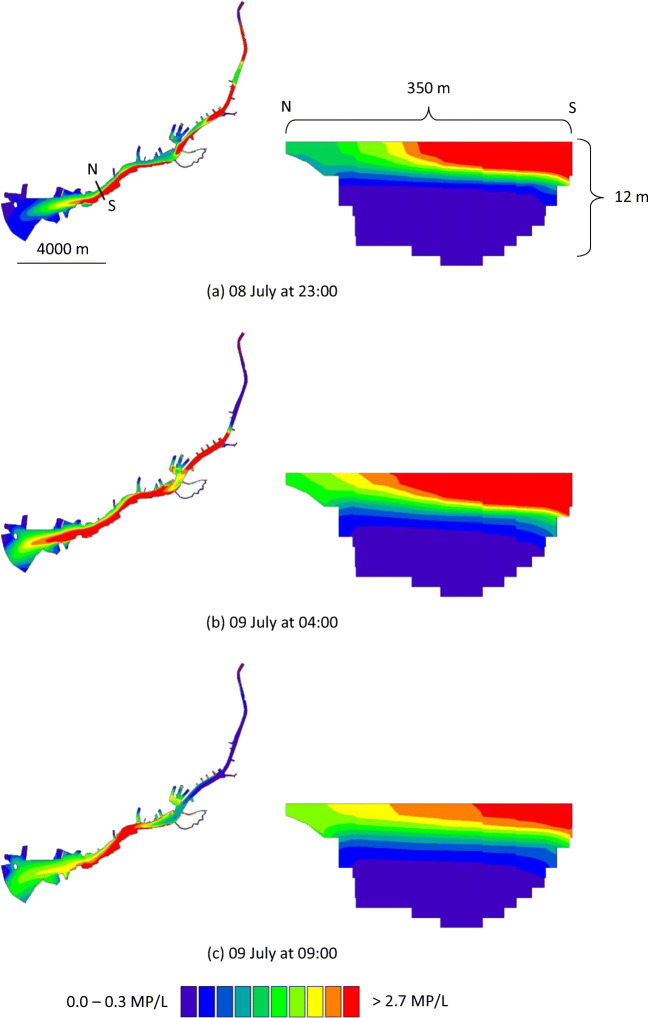
The simulated spread of microplastic particles in the Göta River for Scenario 1 (no settling of particles) at three time points: **a** 8 July at 23:00, **b** 9 July at 04:00 and **c** 9 July at 09:00. The cross-sections (depth up to 12 m) show the concentrations at the Älvsborg Bridge. The location of the Älvsborg Bridge and scale are shown in **a** to the left; the dimensions of the cross-section are shown in **a** to the right

**Fig. 3:**
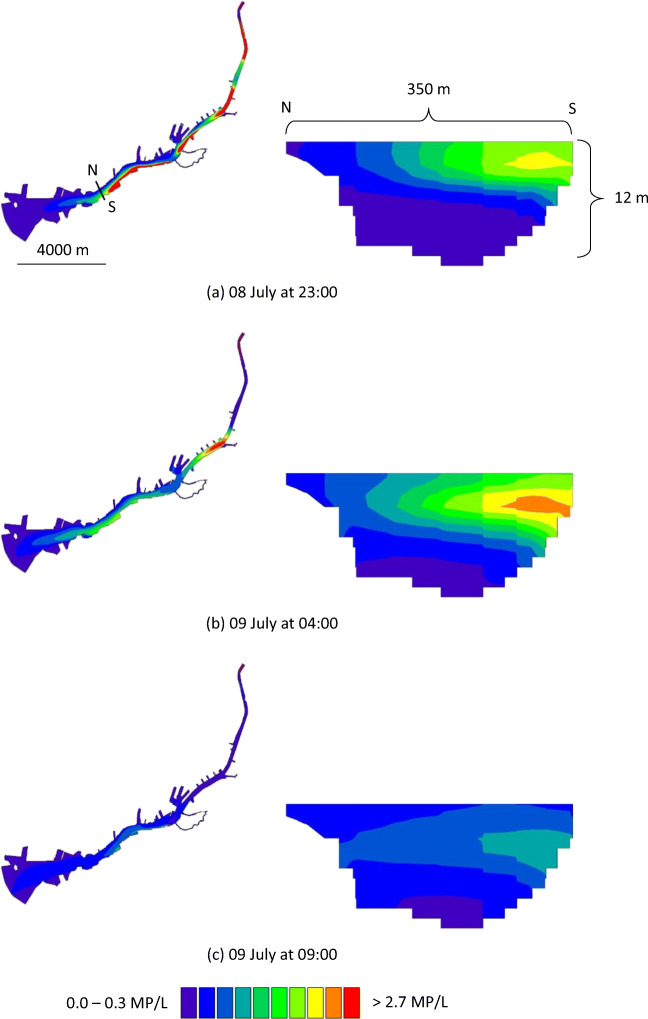
The simulated spread of microplastic particles in the Göta River for Scenario 2 (settling velocity of 1.3∙10^−4^ m/s) at three time points: **a** 8 July at 23:00, **b** 9 July at 04:00 and **c** 9 July at 09:00. The cross-sections (depth up to 12 m) show the concentrations at the Älvsborg Bridge. The location of the Älvsborg Bridge and scale are shown in **a** to the left; the dimensions of the cross-section are shown in **a** to the right

**Fig. 4:**
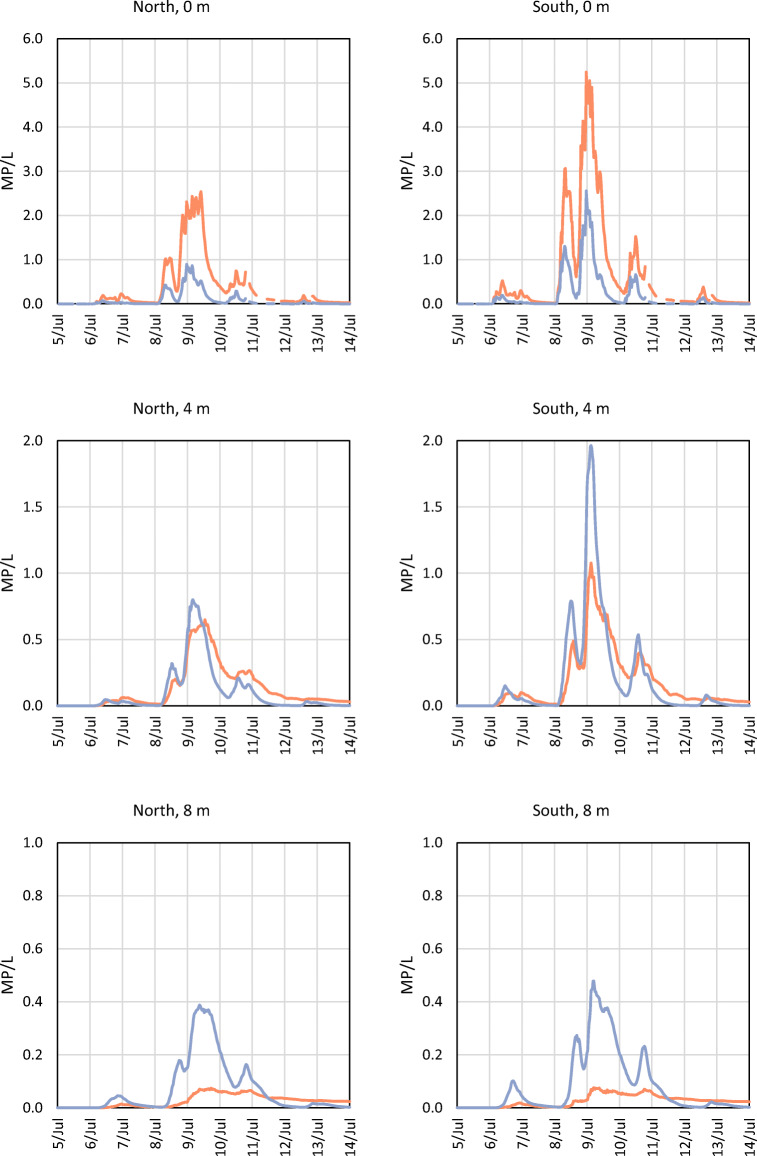
Simulated concentrations of microplastic particles during period 1 (5–13 Jul 2015) at the Älvsborg Bridge at different depths (0, 4 and 8 m) and two different locations across the Göta River (North, 100 m from the north bank of the river, and South, 100 m from the south bank of the river) for Scenario 1 (orange, no settling) and Scenario 2 (blue, settling velocity of 1.3∙10^−4^ m/s)

**Fig. 5:**
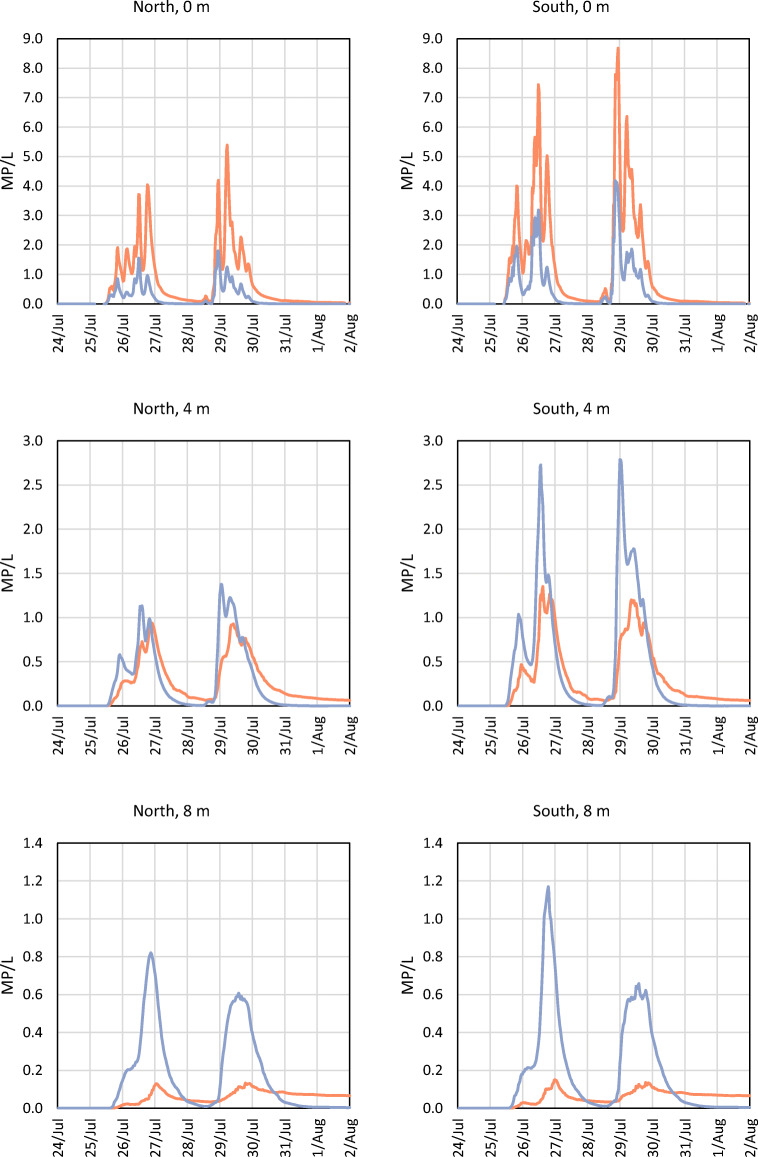
Simulated concentrations of microplastic particles at the Älvsborg Bridge during period 2 (24 Jul–1 Aug 2015) at different depths (0, 4 and 8 m) and two different locations across the Göta River (North, 100 m from the north bank of the river, and South, 100 m from the south bank of the river) for Scenario 1 (orange, no settling) and Scenario 2 (blue, settling velocity of 1.3∙10^−4^ m/s)

The spread of MP in the river was similar during periods 1 and 2 and is illustrated for period 1 (5–13 July 2015) in Figs. 2 and 3. Higher concentrations of MP were found on the south side of the river, compared with the north side. There are more discharge points on the south side of the river, and these points are connected to more heavily polluted areas (e.g. higher annual average daily traffic values; Fig. S8). For Scenario 1 (Fig. 2), the MP concentrations were higher at the water surface than at the deeper levels. It is reasoned that this is due to the release of stormwater at the surface, and the assumption that particles do not settle. For Scenario 2 (Fig. 3), because of settling, particles reached deeper levels in the water column in comparison with Scenario 1. In Figs. 4 and 5, concentrations of MP are shown at the Älvsborg Bridge for different depths (0, 4 and 8 m) and at two locations across the river (100 m from the north bank and 100 m from the south bank of the river) for Scenarios 1 and 2. Again, the higher concentrations of MP at the surface, but not at the deeper levels, for Scenario 1 in comparison with Scenario 2 demonstrated the effect of settling of MP in the river. The results for Scenario 3 are not shown in the figures since they rendered very low MP concentrations. The modelling results showed that the maximum simulated MP concentrations at the Älvsborg Bridge during periods 1 and 2 were 5.2 and 8.7 MP/L, 2.7 and 5.0 MP/L and 2.9∙10^−3^ and 1.4∙10^−2^ MP/L for Scenarios 1, 2 and 3, respectively. The concentrations of MP decreased with depth for both Scenarios 1 and 2 (Figs. 4 and 5). The mixing processes in the river and the MP concentrations were influenced by the vertical water density gradient at the Älvsborg Bridge caused by saline water from the Kattegat strait (Fig. S9). The low concentrations of MP at the Älvsborg Bridge in Scenario 3 implied that most particles settled prior to reaching this location and hence were not transported to the sea in the simulated scenario. However, resuspension was not included in the model, and if this is a dominant process in the river, settled particles can be resuspended into the water column and/or transported with bed sediment to the sea. Larger ships navigate the river, and turbidity levels were seen to increase when certain ships move upstream.

## Discussion

The number of models for MP transport in watersheds and water sources has increased in recent years. There are models based on mass balance and conceptual modelling approaches, which have the advantage that overall transport of MP in large regions can be captured, and high loads of MP entering our oceans can be identified (see, e.g. van Wijnen et al. (2019) and Vermeiren et al. (2016)). However, as stated by van Wijnen et al. (2019), such models are not well equipped to describe local catchments, as they often exclude the effects of the river characteristics, such as flow distribution, river geometry, time dependency and turbulence. In our work, by using a three-dimensional model of the Göta River, the distribution of MP in the river was captured in the length, depth and width directions. The model accounted for the bathymetry of the river, the time-dependent flow regime and the dynamic release of stormwater during the rain events in 2015. To understand how the size and density and thus settling velocity of MP particles affect the distribution of MP in the river during the rain events, settling was incorporated into the model. However, many other factors potentially affecting the fate and transport of MP were not included in the model, and the effects of these factors are further discussed below.

The selected scenarios, in which the settling varied between 0 and 2.4∙10^−3^ m/s, demonstrated the importance of settling velocity assumptions for the modelling results (Figs. 2, 3, 4 and 5). For Scenario 2, the settling velocity assumptions were deemed realistic, i.e. the low settling velocity means that the particles are unlikely to settle within the sewer system and enter the river with stormwater discharges. For Scenario 3, the assumption of higher settling velocity potentially implies that the particles can settle within the sewer system and hence are less likely to enter the river with stormwater discharges. The settling velocities calculated using Stokes’ law are affected by the assumptions of spherical shape of the particles and fresh water. These assumptions were simplifications; in reality, the particles have varying shapes, and saline water from the sea enters the river.

In the modelled scenarios, it was assumed that the MP are inert and that settling alone, in addition to advection-dispersion processes, affect the particle fate in the river. However, MP undergo several processes once released into the aquatic environment, including fragmentation/degradation, biofouling, aggregation, resuspension and/or burial in bottom sediments. Fragmentation of MP is caused by a combination of physical abrasion, photo-oxidation, hydrolysis and degradation by microorganisms (Kooi et al. 2017, 2018; Song et al. 2017). Fragmentation processes would create smaller particles which would settle more slowly. These processes are, however, generally slow and were assumed to take much longer than the residence time of MP in the water column of the Göta River and not considered in the simulations. Similarly, biofouling may affect the settling rate of MP, but the effect was assumed to be small because of the short residence time of MP in the river water and the slow rate of the biofouling process (Fazey and Ryan 2016). Studies on biofouling effects on MP in freshwater systems have increased in the last few years (Harrison et al. 2018; Horton et al. 2017a, b), yet further studies need to be conducted to answer questions regarding the reaction of different types of plastic particles with different environments (e.g. stormwater, receiving freshwater), causes of the different reactivities and effects of local environmental conditions including biological factors. Microplastic particles may also form aggregates with naturally occurring colloids including mineral and biogenic particles such as plankton or other plastic particles (Corcoran 2015; Alimi et al. 2018; Michels et al. 2018). Aggregation increases the settling velocity of MP and is dependent on factors such as particle concentrations, sizes and densities and attachment efficiencies (Kooi et al. 2017, 2018). In Besseling et al. (2017), where aggregation was included in the fate simulation of nano- and microplastics in a freshwater system, it was concluded that aggregation has a larger effect on the settling of nanoplastics, whereas microparticles were rather unaffected. As only particles in the microscale were included in the current study, aggregation was not considered in the simulations. Microplastics can be chemically altered both through leaching of plastic additives, e.g. phthalates, bisphenol A (BPA), alkylphenols, polybrominated diphenyl ethers (PBDEs), which are not chemically bound to the polymer, and through adhering of waterborne pollutants including both metals and organic compounds (Avio et al. 2017; Wright et al. 2013). However, it is currently not clear how these chemical alterations affect the density and the fate of MP in the water column. Particles settled in the top layer of the riverbed can be resuspended if the shear stress of the flowing water is high enough. Annual sediment transport in the Göta River has been estimated to at least 130,000 t, of which approximately 40,000 t is caused by ship-generated waves (Bondelind et al. 2015). However, in the current study, resuspension was not included due to uncertainties in the required parameterisation of this process.

In addition to the uncertainties in MP fate once they are released into receiving waters, very little is known about the processes that affect MP at the emission source and during transport through the stormwater system. In this study, the same MP concentrations in road run-off were assumed for all simulated rainfall events, whereas differences between rainfall events in terms of antecedent dry period and rainfall intensity and duration may result in different particle content in road run-off; these effects can be considered in further studies using build-up/wash-off modelling (Murakami et al. 2004; Egodawatta et al. 2007; Zhao and Li 2013). Also, road salt and sand application for winter road maintenance could increase salinity and particle content of road run-off released into the river. In this study, it was assumed that all MP present in road run-off reach the stormwater discharge point in the river; however, further fragmentation/degradation, biofouling, aggregation and settling in sediment traps, such as ponds and gullies, may affect particle transport in the stormwater system (Vogelsang et al. 2018).

The MP concentrations used in this study (Table 2) were estimated from reported concentrations of MP in stormwater in Gothenburg (Table 1) and the AADT reported by the City of Gothenburg (Göteborgs Stad 2019). Since tyres were assumed to be the largest source of MP in stormwater (Vogelsang et al. 2018; Magnusson et al. 2016; Siegfried et al. 2017), sources other than traffic were neglected. The MP concentration (1050 MP/L), which all discharge concentrations were based on (Table 2), was considered reasonable as it was within the concentration ranges of MP reported in other studies of road run-off (Trafikkontoret Göteborgs Stad 2018; Jönsson 2016). However, more studies of MP in run-off from different types of traffic areas are desirable to understand how traffic patterns, such as vehicle count and speed, affect MP emissions and how road and drainage design affect transport of MP from the road into the stormwater system. The estimated AADT connected to each discharge point is considered uncertain, as the definite boundary of each catchment area was not known. Hence, the AADT for each discharge point was collected from traffic measurement points assumed to be located in the catchment area. It should also be noted that traffic data from different roads and streets were collected in different years (Göteborgs Stad 2019). Consequently, simulated MP concentrations should be considered indicative, and Figs. 2, 3, 4 and 5 should be interpreted as likeliness of particle occurrence at specific vertical and horizontal layers, rather than true MP concentrations.

Although MP concentrations used as input for the simulations were uncertain, the used MP particle size and density were based on more available literature data. Studies reviewed by Vogelsang et al. (2018) reveal that the volume size distribution of tyre-related particles are in the size range of 10–100 μm with an average particle size of 65 to 85 μm. Particles found in stormwater in Gothenburg were of the same size range of 20–100 μm (Trafikkontoret Göteborgs Stad 2018). The density of polymers ranges from below water density (< 1 g/cm^3^), e.g. PE and styrene-butadiene, to over 1.5 g/cm^3^, e.g. PVC and melamine (Avio et al. 2017). Hence, the buoyancy and settling rate of MP particles in aquatic environments may vary considerably. Abrasion of tyres produces particles that are a mix of the tread material and road surface material (Adachi and Tainosho 2004; Kreider et al. 2010). It has been estimated that the tread makes up approximately 40% of tyre wear particles; because the remaining 60% are made up of minerals, the density of tyre wear particles is considerably higher than of the original tread (Vogelsang et al. 2018). This implies that the fate of tyre-related particles in aquatic environments is very different from many of the most commonly used polymers such as PE, PP and PS of lower density (Avio et al. 2017; Hanvey et al. 2017).

To validate the simulations, a monitoring programme of MP concentrations in stormwater discharges and in the Göta River would be needed. Currently, few commercial laboratories offer MP analyses, available analyses are very costly, and standardised procedures for MP quantification and reporting are missing. In this study, river water samples were collected close to discharge point GÄ6 (Fig. 1) at 1 m depth during two rain events in July and August 2018. Plastic particles > 50 μm were analysed using FTIR (Fourier Transform Infrared Spectroscopy). Tyre particles were not analysed, as pyrolysis-GC-MS is required to identify black rubber particles (Gueissaz and Massonnet 2013). In the collected samples, 2–3 plastic particles (fibres and/or fragments) per litre were identified, sizes ranging from 186 to 905 μm (longest axis). The sampling approach (one location, two grab samples of 1 L each from one depth during rain) and analysis (non-rubber particles > 50 μm) encompass many limitations, and from the sampling results, it is not possible to draw any conclusions on MP occurrence in the Göta River as a result of road run-off discharges.

By using hydrodynamic modelling, areas with high concentration of MP can be identified, facilitating the choice and location of mitigation measures. Since traffic is one of the largest contributors to MP in urban environments, preventive actions such as congestion taxes and improved tread composition to reduce MP emissions, as well as technical measures to minimise transport of MP with road run-off, are urgently needed. Although treatment facilities currently used for road run-off are not specifically designed to remove MP, the facilities are usually designed for particle removal. For larger and heavier MP, sedimentation is assumed to be the primary removal mechanism, whereas filtration or sorption may be needed for lighter and smaller particles with low settling rate. However, as Vogelsang et al. (2018) highlight, evidence that MP are removed using treatment facilities for road run-off is generally lacking. Removal of MP through settling and filtration will lead to contaminated sediments and filter media, which will require further treatment before disposal or reuse. Future research should include possibilities to separate and/or degrade MP from contaminated materials.

## Conclusions

In this study, the effect of the size and density of tyre wear particles in road run-off on their fate in the Göta River in Sweden was investigated using a three-dimensional hydrodynamic model on the example of two time periods in 2015.

- Results showed that higher concentrations of MP were found on the south side of the river, compared with the north side, due to more stormwater discharge points and more heavily polluted catchment areas with higher annual average daily traffic loads along the south side of the river.
- At the Älvsborg Bridge, the mixing processes in the river and the MP concentrations were influenced by the vertical water density gradient, caused by intruding saline water from the Kattegat strait.
- For the studied two periods, most MP with size 75 μm and density 1.9 g/cm^3^ (larger and heavier tyre-related particles) settled in the river, about one third of MP with size 20 μm and density 1.7 g/cm^3^ (average sized tyre particles) settled in the river, and small MP with density close to 1.0 g/cm^3^ did not settle in the river and therefore reached the Kattegat strait.

To prevent traffic-related microplastics from entering the environment, measures aiming to reduce emissions of microplastics, as well as technical measures aiming to prevent/reduce the transport of microplastics with road run-off, are necessary. More research is needed to describe the fate and transport of microplastics in the stormwater system, including treatment facilities, i.e. biofouling, aggregation, degradation and/or further fragmentation and settling.

## Electronic supplementary material

ESM 1 (DOCX 3162 kb)
