# Supplementary material for: Hydrodynamic modelling of traffic-related microplastics discharged with stormwater into the Göta River in Sweden
Source: Environ Sci Pollut Res Int. 2020 Apr 18;27(19):24218–30. doi: 10.1007/s11356-020-08637-z (PMC7326794; doi:10.1007/s11356-020-08637-z)
Supplement: Supplementary file 1 — (DOCX 3162 kb) [file 11356_2020_8637_MOESM1_ESM.docx]

**Hydrodynamic modelling of traffic-related microplastics discharged with stormwater into the Göta River in Sweden**

Mia Bondelind^1^, Ekaterina Sokolova^1^*, Ailinh Nguyen^1^, Dick Karlsson^2^, Anna Karlsson^3^, Karin Björklund^1^

^1^ Department of Architecture and Civil Engineering, Chalmers University of Technology, Sven Hultins gata 6, SE-412 96 Gothenburg, Sweden

^2^ Sustainable Waste and Water, City of Gothenburg, Box 123, SE-424 23 Angered, Sweden

^3^ Tyréns AB, Lilla Badhusgatan 2, SE-411 21 Gothenburg, Sweden

*Corresponding author, e-mail: [ekaterina.sokolova@chalmers.se](mailto:ekaterina.sokolova@chalmers.se)

# Supporting information

This supporting information is largely based on two earlier publications, i.e. the report by Tyréns AB (2016) describing the set-up and validation of the hydrodynamic model for the Göta River, and the article by Björklund et al. (2018) reporting on the use of this hydrodynamic model to simulate the concentrations of benzo[a]pyrene and copper in the Göta River:

- Tyréns AB (2016), Badvattenkvalitet Göta älv – modelleringsstudie (Bathing water quality in the Göta River - modelling study). Göteborg, Sweden.
- Björklund, K., Bondelind, M., Karlsson, A., Karlsson, D., Sokolova, E. (2018), Hydrodynamic modelling of the influence of stormwater and combined sewer overflows on receiving water quality: Benzo(a)pyrene and copper risks to recreational water. *Journal of Environmental Management* **207**, 32-42.


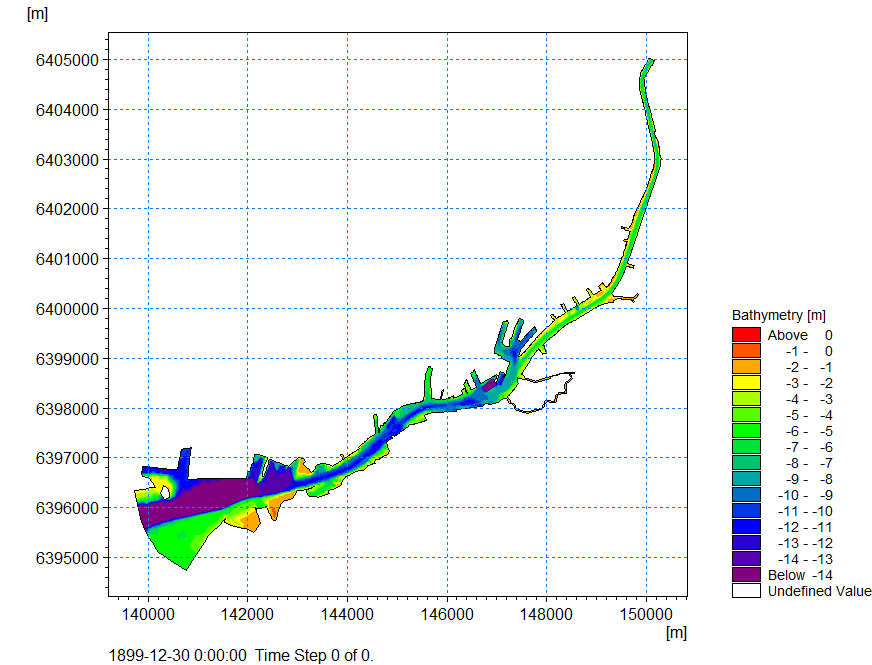


**Fig. S1** Bathymetry of the simulated stretch of the Göta River (data from map 931 by the Swedish Maritime Administration and high resolution multi-beam measurements performed in 2009 for the Port of Gothenburg and the City of Gothenburg).

In the hydrodynamic model, data on the water flow (Fig. S2 and S3) were used to specify the conditions at the upstream boundary and in the tributaries, and data on the water level (Fig. S2) were used to specify the conditions at the downstream boundary. The initial conditions in the river were specified as constant surface elevation.

**Fig. S2** Water flow in the studied stretch of the Göta River (data from Vattenfall) and water level at the Kattegat strait during the studied period in 2015 (data from the Swedish Meteorological and Hydrological Institute, station Torshamnen).

**Fig. S3** Water flow in the tributaries within the studied stretch of the Göta River during the studied period in 2015 (data from Vattenwebb by the Swedish Meteorological and Hydrological Institute, <http://vattenwebb.smhi.se/>).

**Fig. S4** Wind speed and direction during the studied period in 2015 (data from the Swedish Meteorological and Hydrological Institute, station Göteborg A located at 57°42’56.4”N 11°59’33.0”E).

In the model for the studied period 5 – 13 July 2015, the conditions for temperature and salinity at the upstream boundary and in the tributaries were specified as constant in time and in space: 16 °C and 0 psu, respectively. At the downstream boundary, the conditions for temperature and salinity were specified as constant in time and varying in space according to Table S1. The initial conditions were described as constant in space: 16 °C and 0 psu.

**Table S1** Measurements of salinity and temperature in the Kattegat strait on 3 August 2015 (data from the Water Management Association of the Bohus Coast, station Skalkorgarna).

| Depth, m | 0 | 2 | 5 | 10 | 14 |
| --- | --- | --- | --- | --- | --- |
| Salinity, psu | 12.52 | 19.35 | 22.29 | 22.83 | 23.01 |
| Temperature, °C | 17.5 | 17.1 | 16.6 | 16.6 | 16.4 |

The hydrodynamic model of the Göta River was validated by comparing the simulated and measured salinity profiles in the river (Fig. S5).


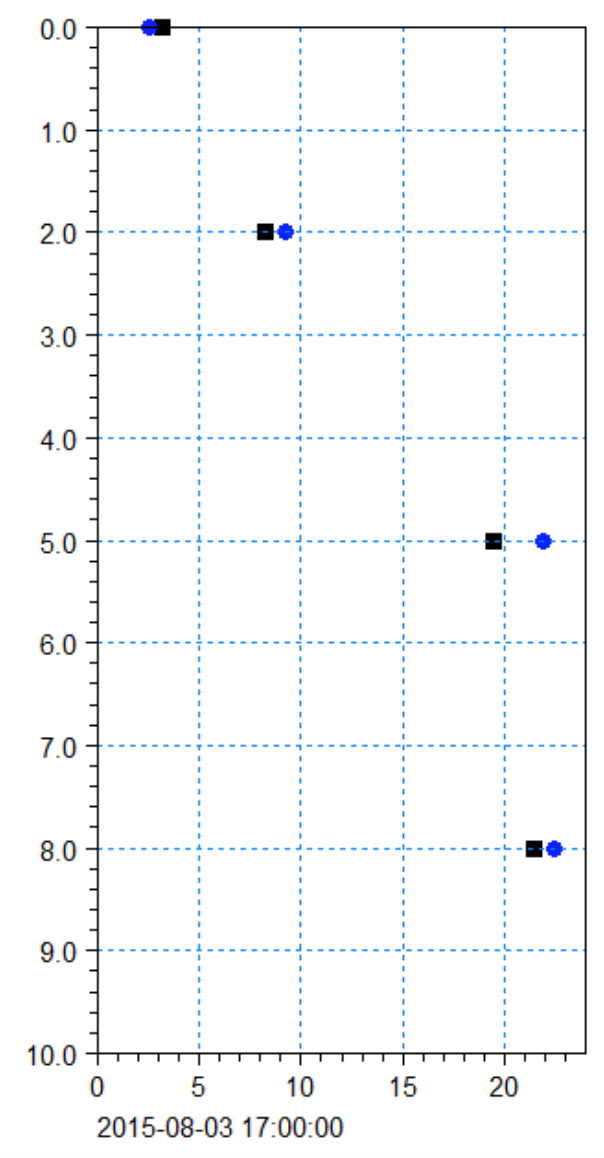


Salinity, psu

Depth, m

**Fig. S5** Comparison of the simulated (blue dots) and measured (black dots) salinity in the Göta River on 3 August 2015 (data from the Water Management Association of the Bohus Coast, station Älvsborgsbron). The figure is reproduced from: Tyréns AB (2016) Badvattenkvalitet Göta älv – modelleringsstudie (Bathing water quality in the Göta River - modelling study); Kretslopp och vatten, Göteborgs stad, Gothenburg, Sweden.

A)

B)

**Fig. S6** Precipitation intensity (mm/h) during the simulated periods: A) 5 – 13 Jul 2015 and B) 24 Jul – 1 Aug 2015. The annual average precipitation in Gothenburg is 855 mm. The total precipitation during 2015 was 1038 mm. All data are taken from the Swedish Meteorological and Hydrological Institute: <https://opendata-download-metobs.smhi.se/explore/?parameter=3>. The rain gauge is located at 57°42’56.4”N 11°59’33.0”E.

**Fig. S7** Stormwater discharges into the studied stretch of the Göta River during the studied period in 2015 (estimations by the City of Gothenburg).

**Fig. S8** Calculated emission of MP with stormwater discharges into the studied stretch of the Göta River during the year 2015: blue – MP emission, orange –stormwater volume.


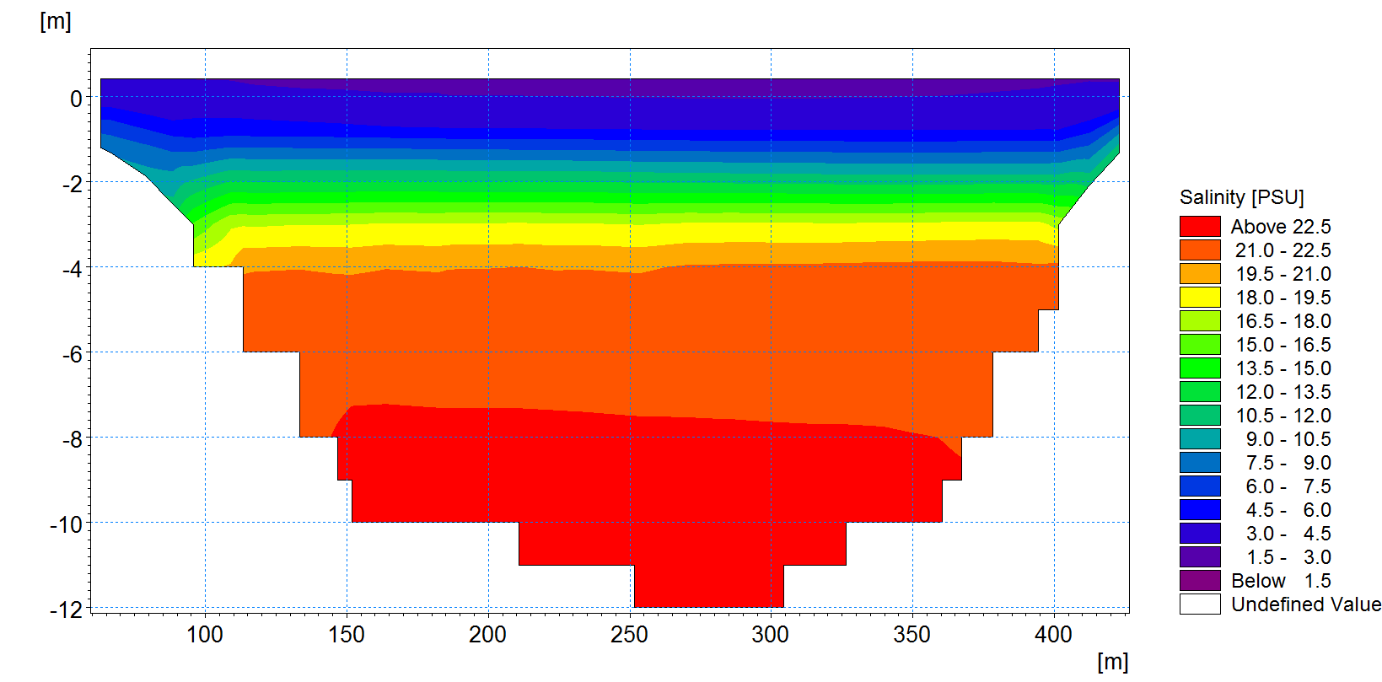


**Fig. S9** Simulated salinity (cross-section) at the Älvsborg Bridge on 9 Jul 2015 at 09:00.
